# Observations on Dr. Harrison's Intended Bill

**Published:** 1811-02

**Authors:** 


*To the Editors of the Medical and Physical Journal.*

GENTLEMEN,

HAVING read a criticism of Dr. Harrison's "Address to the Lincolnshire Benevolent Medical Society" in your last Number, I beg leave, through the same channel, to offer a few remarks upon a subject vitally interesting to the Faculty, and their patients. I do it the more readily, because you have liberally offered to make your valuable Journal the medium of any temperate observations, whether they be favourable to reform or otherwise. There is every reason to expect, as I have repeatedly heard in conversation, that the bill, of which a copious abstract is printed, with Dr. H.'s address, will under certain modifications be presented to the legislature *early in the ensuing Session of Parliament*, and therefore, it behoves every practitioner, anxious for the honour of his art, and the public weal in what relates to it, to examine the bill and address, which includes a comment upon the bill, dispassionately and without pre-conceived notions. Let him in so doing weigh rather what is practicable in reform, than what he himself conceives to be most desirable or agreeable to his private interests.

The reviewer of Dr. Harrison's work observes, "it appears to us, that he (the Dr.) has attempted to lop some misshapen branches of an old tree, whilst he feared to apply the axe to the decayed trunk." A commentator on the same work, in another Medical Journal, cries out, "that the rusty musty corporations must be destroyed." Without offering any opinion of my own on this delicate affair, I think

---

enough enough is stated by your reviewer, to shew the impossibility of overturning these antient fabrics. "The College of Physicians," he observes, "was instituted by royal authority, and from the nature of its constitution is intimately connected with our two English Universities, and consequently may be regarded as forming part of the aristocratic establishment of the country. Any attempt at reform, therefore, not sanctioned by the College itself, must be hopeless, whilst these institutions continue to be upheld by Government." If then it be deemed so difficult to subvert a single college, can it be conceived possible by one effort to pull down nine Medical Corporations, founded on Acts of Parliament, and protected, as many of them are, by royal charters? The very experiment in these unsettled times would savour of insanity. Dr. Harrison, convinced of its futility, has contrived a bill, which bears no reference to the constitution of the public bodies. The question between the Fellows and Licentiates of the College of Physicians, and the examiners and private members of the College of Surgeons, is therefore left wholly untouched.

Loud and reiterated lamentations are likewise made about quack medicines. Every person tolerably acquainted with human nature, and the state of medical affairs, must feelingly deplore the encouragements afforded to these pernicious articles of finance. Much as I disapprove the policy of Government in this respect, and sincerely as I join the malcontents, I must nevertheless be allowed to think it an act of justifiable caution, on the present occasion, to disregard the patent warehouse. Whenever quack medicines are brought under parliamentary review, let them be introduced in a separate bill and undergo a specific investigation, or no good will be done by the attempt. Look at the popular torrent as it now runs in favour of the Eau Medicinale. Do we not discover Peers and Commoners of distinguished talents and political influence, among its decided partizans? Such is the unaccountable rage for this newly imported secret, that no less an individual than the President of that learned and scientific institution, the Royal Society of London, a severe and crippled Podagric, has repeatedly tried its powers and declaims openly in its favor. What is perhaps still more to our purpose, the most fashionable physicians do not hesitate to recommend it, and the King's Sergeant Surgeon has, it is said, condescended to swallow the remedy and to give testimony to its sanative virtues, on his own person. During the continuance of such prejudices with respect to one reputed panacea, can it be reasonably expected that a general clause to restrain the sale of all quack medicines would be favourably received in the British senate? Surely not. Although though corporate societies and empirical nostrums are unassailable, may we not be permitted to try a remedy for the other evils and grievances, under which we have long and confessedly suffered? Dr. Harrison seems to have been actuated by these considerations, when he observes, that "influenced therefore by prudential motives, it was determined to exclude the mention of some less important or objectionable abuses, rather than hazard the whole scheme by making it too difficult and complex, leaving it to the wisdom of after times to correct them, by such provisions as may be suggested by sound policy and subsequent reflection.

It is matter of notoriety, that the provinces and even the capital itself are infested with physicians, surgeons, apothecaries, and venders of drugs, who are acting *as regular persons*, without having passed through a prescribed course of study, or giving any test of professional skill. And are these irregularities to be patiently endured in a civilized country? Shall such an outrage upon society, such a scandal to well informed practitioners be allowed to continue, and no effort made by us to remove the disgrace. Let a becoming regard for the best interests of the people, and for the honour of an useful profession, rouse to immediate exertion. The first process should, I think, be limited to the *regular establishment*. When that has been purified, and made fully competent to its important duties, ulterior regulations will follow in course; because their necessity will become apparent. With this intention, as I conceive, the bill has been limited chiefly to the education and admission of future candidates, leaving a great variety of other matters wholly unnoticed. From the agitated and suspicious temper of the faculty, it appears that Dr. Harrison judged well, in confining his first efforts to a few points. The different orders of the profession are so extremely jealous of each other, that it is next to impossible to devise any measure likely to meet with general approbation. In different conversations with Dr. Harrison, while in London, last summer, I found his chief expectations are from parliament. He was admitted to an official interview with the Chancellor of the Exchequer, and had communications with many leading persons in both Houses. A privy counsellor, one of the most active members of the government, would have introduced the bill into the House of Commons had he received it early enough in the late sessions. A peer, who is of the *cabinet*, has also voluntarily offered his best services. This nobleman, *like many others*, thought the present measure insufficient for an effectual arrangement of the profession. Being told *what subsequent matters are in contemplation*, he declared his his entire approbation, and his determination to support the bill.

Provision is made in the bill not only for the passing generation to continue their functions unmolested, but to confirm them *in the legal enjoyment of their several practices*, consequently the plan will be highly beneficial to the present establishment. Unadmitted persons are required to submit to certain preliminary conditions, which in fact were meant to have been included in the several acts of Henry VIII. In his reign the colleges of physicians and surgeons were constructed to examine and admit all physicians and surgeons. The company of apothecaries was afterwards founded to superintend the practice of pharmacy. The present bill professes to supply these defects, and to provide for the introduction of midwives, and all venders of drugs. It is no where declared that the scheme offered to the medical public is yet complete, or divested of formidable objections. The friends readily admit its imperfections, and are solicitous to receive the candid remarks of well informed individuals, that its defects may be supplied, before the sitting of parliament. In stating difficulties or offering corrections, gentlemen are particularly requested to confine their remarks to what is contained in the bill itself, since they cannot be acquainted with the nature or extent of all that is intended. I may safely assert from what I have heard, that the matters brought forward by your several correspondents, and other irregularities not noticed by them, are well known and under consideration. I may further add, that some verbal alterations have been suggested to the solicitors: *e. g.* In the clause for physicians "to receive" is wholly omitted. In that for surgeons "to claim" is substituted for "to receive." By these trifling alterations, it is believed, that the utmost degree of latitude possible will be given to surgeons in the practice of physic, and to apothecaries in both physic and surgery.

The bill seems in one material instance to have been generally misunderstood, and in consequence to have been unjustly abused. An opinion seems to prevail, that the bill will forcibly restrain physicians, surgeons, and apothecaries to their respective lines. No such thing was ever in contemplation. Dr. Harrison, to tranquillize the apprehensions of the profession, declares in his pamphlet, "in order to remove the fears, and correct the mistakes of some people, it may be proper to observe, that the bill does not attempt to limit the sphere of medical duty by coercive statutes.* Practitioners will be

* See Dr. Harrison's "Address," &c. p. 87.

left left *under it at full liberty to use their talents according to their own discretion.* It will however oblige all future medical men to pass through a suitable course of study, and undergo examinations for the particular branch, or branches of the profession, into which they are admitted. If any of these persons be desirous afterwards to act beyond the authority of his Diploma, as is now the case with many in the profession, and can succeed with the public by his address or merit, he will not be prevented by the bill from following the bent of his inclination." From Dr. Harrison's frequent consultations with the Solicitor, and the Barrister, who drew out the bill, *we may fairly conclude, that he is fully authorized in making the above statement.* The action lately brought by Messrs. Price and Thompson in the Common Pleas, to recover for medicines and attendance, affords sufficient proof that surgeons and apothecaries are not legally empowered to charge *for medical attendance.* From the declaration of the Chief Justice, and the reasonableness of the thing, I trust, if we can ever lay the foundation of reform, that something will be done to increase the receipts of apothecaries, *whose incessant labours are so ill requited,* and who, in order to obtain a miserable pittance, are obliged to drench their employers with supernumerary medicines. It must have struck every observer, that in small towns and sequestered spots, the rich as well as the poor must continue to place their reliance, as heretofore, in apothecaries, because the emoluments will neither admit of division, nor offer sufficient encouragement to induce physicians, or surgeons, to settle there. Had the scheme prevented apothecaries from exercising physic or surgery, it would have increased the distresses of invalids instead of alleviating them. I am the more desirous to correct this misconception, that the faculty of every denomination may fully understand the extent and tendency of the proposed act. Should any person remain sceptical after this explanation, he will I think do well to state his difficulties, and have the sanction of some legal practitioner, before he ventures hastily and inconsiderately to oppose and condemn clauses, which have been framed by a *Barrister of the first eminence in this department of law,* and have been unequivocally approved by some of the most respectable physicians, surgeons, apothecaries, and accoucheurs of the present age.

I am, Gentlemen,

Your very obedient humble Servant,

H. R.

*London, Dec.* 14, 1810.

